# Gyrification patterns in first-episode, drug-naïve major depression: Associations with plasma levels of brain-derived neurotrophic factor and psychiatric symptoms

**DOI:** 10.3389/fpsyt.2022.1031386

**Published:** 2023-01-06

**Authors:** Tomoya Natsuyama, Naomichi Okamoto, Keita Watanabe, Enkhmurun Chibaatar, Hirofumi Tesen, Gaku Hayasaki, Atsuko Ikenouchi, Shingo Kakeda, Reiji Yoshimura

**Affiliations:** ^1^Department of Psychiatry, University of Occupational and Environmental Health, Kitakyushu, Japan; ^2^Open Innovation Institute, Kyoto University, Kyoto, Japan; ^3^Medical Center for Dementia, Hospital of University of Occupational and Environmental Health, Kitakyushu, Japan; ^4^Department of Radiology, Graduate School of Medicine, Hirosaki University, Hirosaki, Japan

**Keywords:** gyrification, major depression, brain-derived neurotrophic factor, gray matter, cerebral cortex

## Abstract

**Background and objectives:**

Cortical structural changes in major depressive disorder (MDD) are usually studied using a voxel-based morphometry approach to delineate the cortical gray matter volume. Among cortical structures, gyrification patterns are considered a relatively stable indicator. In this study, we investigated differences in gyrification patterns between MDD patients and healthy controls (HCs) and explored the association of gyrification patterns with plasma brain-derived neurotrophic factor (BDNF) levels and depressive symptoms in MDD patients.

**Methods:**

We evaluated 79 MDD patients and 94 HCs and assessed depression severity in the patients using the 17-item Hamilton Depression Rating Scale (HAM-D). Blood samples of both groups were collected to measure plasma BDNF levels. Magnetic resonance imaging (MRI) data were obtained using three-dimensional fast-spoiled gradient-recalled acquisition. Differences in plasma BDNF levels between groups were examined using the Mann–Whitney U test. Principal component analysis and orthogonal partial least squares discriminant analysis (OPLS-DA) were conducted to investigate the gyrification patterns which were significantly different between the groups, i.e., those with variable importance in projection (VIP) scores of >1.5 and *p*-value < 0.05 in multiple regression analyses adjusted for age and sex. Finally, multiple regression analysis was performed on the selected gyrification patterns to examine their association with BDNF levels in the two groups and HAM-D in the patients.

**Results:**

There were no significant differences in plasma BDNF levels between the groups. We found that 108 (71.0%) of 152 total local gyrification indices were MDD < HC. We identified 10 disease-differentiating factors based on critical gyrification features (VIP > 1.5 and *p*-value adjusted for age and sex < 0.05). However, we found no significant correlations between the 10 gyrification patterns and plasma BDNF levels and no interaction with group. Moreover, no significant correlations were observed between the local gyrification indices and HAM-D total scores.

**Conclusion:**

These results suggest that abnormal early cortical neurodevelopment may mediate vulnerability to MDD, independent of plasma BDNF levels and depressive symptoms.

## Introduction

Major depressive disorder (MDD) is a highly prevalent and debilitating mental disorder that affects more than 264 million people worldwide (1). Individuals with depression commonly experience dysfunctional symptoms, including undesirable mood, impaired concentration, and poor sleep quality, and—more importantly—these patients are at high risk (up to 15%) of suicide (2). Moreover, previous evidence shows that MDD is a risk factor for physical illnesses (3), and the most common comorbidities are chronic physical conditions such as cardiovascular and respiratory diseases, diabetes, arthritis, osteoporosis, and cancer (4). MDD is predicted to become the second major contributor to the general medical service burden by 2030 (5, 6). The pathophysiology of MDD is complex and no single model or mechanism can fully explain it. Growth and adaptation at the neuronal level is more broadly termed neuroplasticity, and it is presumably this cellular-level neuroplasticity that is altered by inflammation and hypothalamus-pituitary-adrenal axis dysfunction due to environmental stress. The process of neurogenesis is controlled by regulatory proteins. Animal studies have shown that limiting neurogenesis inhibits antidepressant effects and produces depression-like symptoms, especially in stressful situations. Thus, it has been suggested that neurogenesis promotes resilience to stress, which may be the basis for the clinical efficacy of antidepressants. Postmortem studies of MDD patients have demonstrated a loss of granule neurons in the dentate gyrus of untreated patients compared to treated and non-MDD patients. There are considerably more splitting neural progenitor cells in patients treated for MDD compared to untreated MDD and even non-MDD patients. These findings are consistent with mouse studies showing that antidepressants act by increasing neurogenesis in the adult brain. In short, neuroplasticity plays an important role in the pathophysiology of MDD (7, 8).

Cortical gyrification, a forming process of cerebral cortex folds, begins between 10 and 15 weeks of fetal development. Cortical folding related to fetal and early postnatal neurodevelopmental processes is defined by the local gyrification index (LGI) (9). LGI quantifies the amount of cortex buried within the sulcal folds and represents the extent of cortical folding. It is expressed as the ratio of the entire outer cortical surface (superficial exposed area plus the area buried in the sulcus) to the superficial exposed area (10, 11). The index of each vertex is calculated by dividing the ossicular surface area by the corresponding outer surface area (12). LGI increases dramatically during the third trimester of pregnancy and peaks by age 2, then remains relatively constant throughout the rest of life (13–15). Cortical thickness and surface area mainly characterize the neuronal density and number and spacing of the cortical columns, respectively (16–18). Therefore, computing LGI, the pattern and degree of cortical folding, might be a more stable indicator for investigating abnormal neurodevelopmental patterns in MDD. Additionally, recent technological developments in 3D image reconstruction and surface morphometry have improved LGI measurement and gained wide acceptance in psychiatric research (19).

Voxel-based morphometry is a frequently used approach in MDD patients to assay cortical structural changes and to delineate the cortical gray matter volume. Considering many ongoing studies, researchers have identified several neuroanatomical changes in MDD, including widespread focal alterations in cortical thickness (20), surface area (21), and cortical gyrification (9). A large-scale meta-analysis demonstrated distributed cortical alterations that affected the orbitofrontal cortex, anterior and posterior cingulate cortex, insula, and temporal lobes (21). Additionally, MDD is considered a disorder of dysregulated neural networks, including irregularities in the neural connection (22, 23), rather than regional abnormalities (24). Thus, direct investigation of cortical thickness, surface area, and folding patterns—which reflect different biological factors—is better and more accurate in revealing the structural alterations in MDD (14, 15). Cortical folding related to fetal and early postnatal neurodevelopmental processes is defined by LGI (9, 10, 25, 26). Thus, computing LGI might be ideal for investigating abnormal neurodevelopmental patterns in MDD. Several studies have found different LGI in patients with MDD than in healthy controls (HCs) (27–29). Reports on changes in LGI in patients with MDD are inconsistent (9, 27–29), but have not largely explored the association with depressive symptoms.

Brain-derived neurotrophic factor (BDNF) is a neurotrophic factor that is essential for neuronal survival, growth, and maintenance in brain circuits involved in emotional and cognitive function (30). Several previous studies and meta-analyses have proved that BDNF is associated with brain neuroplasticity and is involved in the pathophysiology of MDD (31–33). Previous studies also demonstrated decreased plasma or serum levels of BDNF in patients with MDD (34, 35). Perhaps, even more important is the fact that reduced BDNF levels in patients with MDD can be restored by antidepressant therapies such as pharmacotherapy and psychological interventions. Although the relevance of BDNF for MDD is obvious, no studies have investigated the relationship between LGI and BDNF levels, which might be associated with synaptogenesis and neurogenesis. Moreover, recent reports demonstrated that the BDNF gene (Val66Met) was related to changes in gyrification in bipolar disorder (36). Another report indicated that autism spectrum disorder is associated with a distorted relationship between the Val66Met genotype and determinants of regional cortical surface area—cortical gyrification and/or sulcal positioning (37). From these previous findings, it can be postulated that BDNF secretion associated with Val66Met polymorphism may be related to cortical gyrification.

Thus, we investigated differences in gyrification patterns between first-episode, drug-naïve MDD patients and HCs. Moreover, we explored the relationship between cortical gyrification and plasma BDNF levels, which might be associated with synaptogenesis and neurogenesis in both groups. We also explored the association between gyrification patterns and depressive symptoms in patients with MDD based on HAM-D scores.

## Materials and methods

### Participants

Patients were recruited from the Hospital of University of Occupational and Environmental Health in Kitakyushu, Japan. We conducted the full Structured Clinical Interview from the Diagnostic and Statistical Manual for Mental Disorders-5 (DSM-5) (38) for all participants to diagnose MDD and ensure that HCs did not currently fulfill the criteria for any psychiatric diseases. We also ensured that no HC had a history of serious medical and neurological diseases or a family history of major psychiatric or neurological diseases among their first-degree relatives. Although our study population overlaps in part with several of our previously published studies (39, 40), no study has analyzed the correlation between cortical gyrification, plasma BDNF levels, and psychiatric symptoms.

### Clinical assessment and blood sampling

Depression severity was assessed using the 17-item Hamilton Depression Rating Scale (HAM-D) (41). A veteran psychiatrist with 35 years of clinical psychiatric experience assessed patients with MDD using a GRID-Hamilton Rating Scale for Depression (GRID-HAM-D-17 and −21) (42). Patients’ blood samples were collected in regular tubes between 9:00 and 11:00 AM, and plasma samples were separated by centrifugation at 2000 rpm for 20 min. The separated plasma samples were stored at −80°C in silicone-coated tubes until analysis.

### MRI acquisition

Magnetic resonance imaging data were obtained using a 3T MR system (Signa EXCITE 3T; GE Healthcare, Chicago, IL, US) with an 8-channel brain phased-array coil. Images were acquired using three-dimensional fast-spoiled gradient-recalled acquisition. The acquisition parameters were as follows: repetition time/echo time, 10/4.1 ms; flip angle, 10°; field of view, 24 cm; and resolution, 0.9 × 0.9 × 1.2 mm. All images were corrected for distortion due to gradient non-linearity using the “Grad Warp” software program.

### Measurement of gyrification

To measure gyrification, the Statistical Parametric Mapping 12 (SPM12)^1^ and computational anatomy toolbox 12 (CAT12)^2^ were used. The preset parameters for the processing and analysis steps were under the CAT12 user manual.^3^ We then measured the local gyrification indices (LGIs) of 152 cortical regions from the apart a2009s segmentation (43).

### Statistical analysis

All statistical analyses were performed using R and Python 3.0. We examined the differences in plasma BDNF levels using the Mann–Whitney U test. We also conducted a principal component analysis (PCA) and plotted a two-dimensional plot for differences in LGIs between MDD and HC groups. Next, we performed orthogonal partial least squares discriminant analysis (OPLS-DA) to obtain a further separation (44). OPLS-DA is a type of discriminant analysis, introduced as an improved version of partial least squares discriminant analysis (PLS-DA), which uses multivariate data to discriminate two or more groups. It quantitatively determines which variables contribute to the separation of groups. Each symbol in OPLS model overview represents the following value: dataset [X], class labels [Y], predictability of the models [Q^2^], goodness of fit of the model [R^2^], predictive component [p1], the first orthogonal component [o1]. Based on the OPLS-DA, we selected specific gyrification patterns that characterized the differences between MDD and HC groups with variable importance in projection (VIP) score of >1.5 (45). A VIP score is a measure of a variable’s importance in the PLS-DA model. It summarizes the contribution of a variable to the model. The VIP score of a variable is calculated as a weighted sum of the squared correlations between the PLS-DA components and original variable. In conjunction with the VIP score, we calculated *p*-value for differences in gyrification patterns between MDD and HC groups using Welch’s *t*-test and multiple regression analysis adjusted for age and sex. Multiple regression analysis was then performed on the selected gyrification patterns to examine their association with BDNF level and HAM-D. In addition, we used multiple regression analysis to test the interaction between groups and BDNF level.

The distributions of all data were confirmed using histograms and expressed as mean (standard deviation) or median [interquartile range]. In the multiple regression analysis, the normality of residuals was checked to confirm the model’s validity. Missing data were excluded from the analysis. The test was two-tailed, with a *p*-value of <0.05 set as statistically significant. For correlation analyses, Bonferroni corrections were performed for those that reached statistical significance.

### Ethical considerations

This study was conducted in accordance with the principles of the Declaration of Helsinki, and the relevant Ethics Committee approved the protocol. Informed consent was obtained from all participants, and we assigned each patient an arbitrary identification number to protect their privacy.

## Results

### Demographic data and clinical characteristics of participants

Demographic and clinical characteristics are shown in Table 1. Differences in age and sex were observed.

**TABLE 1:** Demographic and clinical characteristics data.

Demographic data	HC (*n* = 94)	MDD (*n* = 79)	*p*-value
Age	33 [28–42.5]	54 [42–67.5]	<0.001
Sex (male, %)	53 (67.1%)	35 (44.3%)	0.006
Dominant arm (right, %)	52 (100%)	35 (94.6%)	0.17
Duration of illness (months)	–	3 [1.5–6.5]	–
HAM-D scores	–	21 [18–27]	–

HAM-D, Hamilton Depression Rating Scale; HC, healthy control; MDD, major depressive disorder.

### LGIs in MDD and HC groups

**FIGURE 1:**
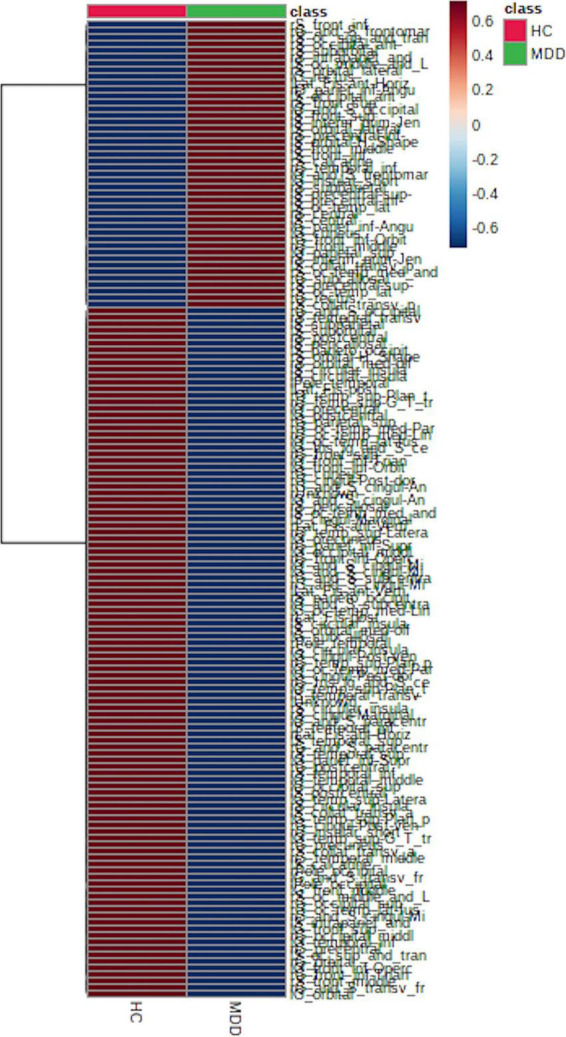
Local gyrification index (LGI) between major depressive disorder (MDD) and healthy control (HC) groups. We found that 108 (approximately 71%) of the 152 LGIs were MDD < HC, which supports a decrease in LGIs in patients with MDD.

LGIs in MDD and HC groups are shown in Figure 1 and Supplementary Table 1; 108 (71.0%) of the 152 LGIs measured in this study were MDD < HC.

### Differences in plasma BDNF levels between MDD and HC groups

The median plasma BDNF level in the MDD group was 5.0 ng/mL [2.65–7.95], while the median in the HC group was 3.8 ng/mL [2.12–8.25]. There was no significant difference in plasma BDNF levels between the two groups (*p* = 0.35).

### PCA and OPLS-DA

**FIGURE 2:**
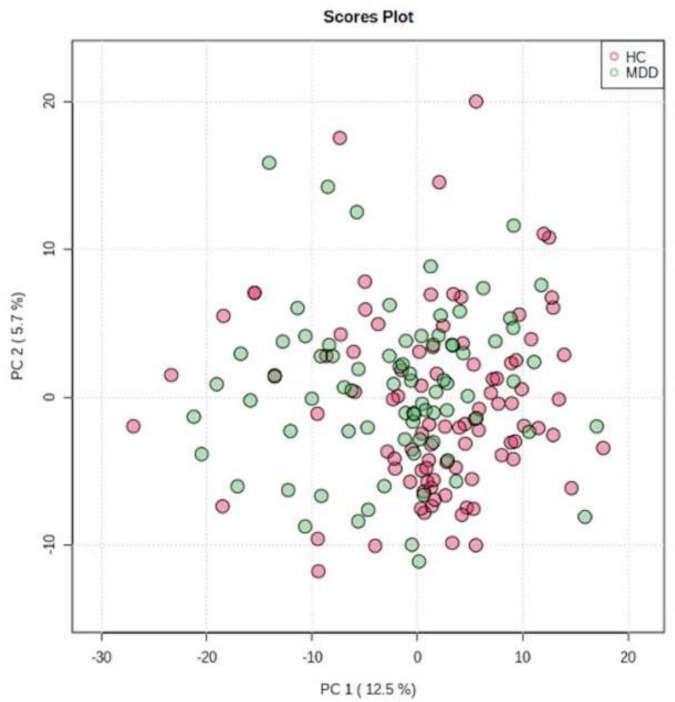
Principal component analysis (PCA). We evaluated all LGIs using PCA and created two-dimensional plots for the first and second principal components.

We evaluated all LGIs using PCA and created two-dimensional plots for the first and second principal components. PCA separated MDD and HC groups by the first main component, as shown in Figure 2.

**FIGURE 3:**
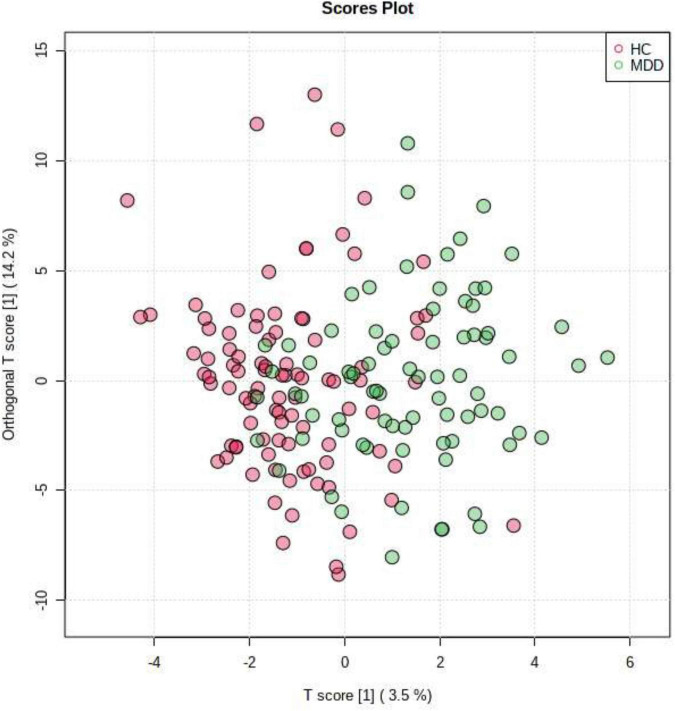
Orthogonal partial least squares discriminant analysis (OPLS-DA). OPLS-DA showed a relatively clear separation from PCA between patients in MDD group and HC group by the first principal component.

To obtain further separation, we evaluated all LGIs using OPLS-DA and created two-dimensional plots. OPLS-DA showed a relatively clear separation from PCA between MDD and HC groups by the first principal component (Figure 3). OPLS model overview was as follows: p1 (R^2^X: 0.035, R^2^Y: 0.195, Q^2^: 0.019); o1(R^2^X: 0.142, R^2^Y: 0.238, Q^2^: −0.0307).

**FIGURE 4:**
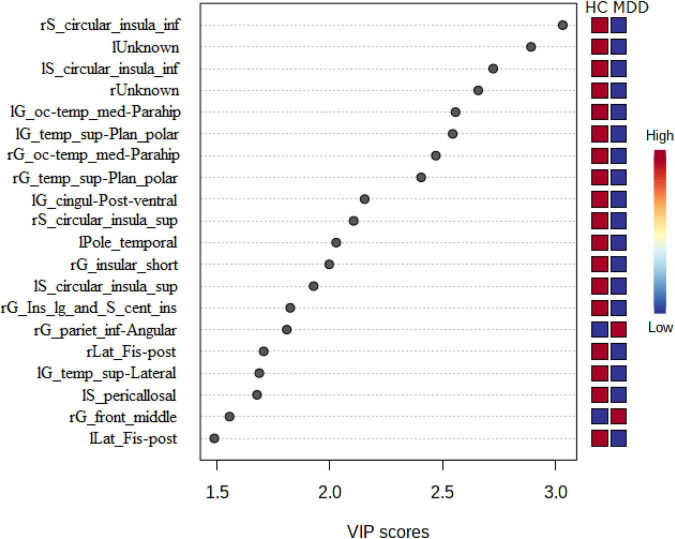
Relationship between expressed LGIs in MDD and HC groups. We showed the 20 significant features of differently expressed LGIs (VIP > 1.5) in MDD and HC groups. “l” and “r” attached to the beginning of the word mean “left” and “right”.

Variable importance in projection scores were extracted from this model (45). Essential features of differentially expressed LGIs were defined as VIP > 1.5. Differentially expressed LGIs in MDD and HC groups are shown in Figure 4 and Table 2. We finally detected 10 differentially expressed LGIs (VIP > 1.5 and *p*-value adjusted for age and sex < 0.05) between HC and MDD (i.e., rS_circular_insula_inf, lUnknown, lS_circular_insula_inf, rUnknown, lG_oc-temp_med-Parahip, lG_temp_sup-Plan_polar, rG_oc-temp_med-Parahip, rG_temp _sup-Plan_polar, lG_cingul-Post-ventral, and rG_insular_ short).

**TABLE 2:** Top 20 gyrification patterns (VIP > 1.5) in major depressive disorder (MDD) and healthy control (HC) groups.

	HC	MDD	*p*-value	Adjusted *p*-value
rS_circular_insula_inf	24.85 (2.43)	23.67 (2.51)	0.002	0.032
lUnknown	23.66 (1.37)	22.95 (1.36)	0.00089	0.037
lS_circular_insula_inf	25.70 (2.51)	24.31 (2.89)	0.00103	0.003
rUnknown	23.32 (1.45)	22.65 (1.27)	0.00153	0.035
lG_oc-temp_med-Parahip	25.64 (1.60)	24.74 (1.66)	0.000452	<0.001
lG_temp_sup-Plan_polar	22.58 (3.55)	20.83 (2.87)	0.000443	0.009
rG_oc-temp_med-Parahip	26.18 (1.62)	25.10 (1.50)	0.0000101	0.001
rG_temp_sup-Plan_polar	23.59 (3.17)	22.07 (2.82)	0.00109	0.005
lG_cingul-Post-ventral	28.7 (3.28)	27.08 (3.84)	0.00291	0.023
rS_circular_insula_sup	29.34 (1.82)	28.81 (1.71)	0.0514	0.15
lPole_temporal	28.56 (1.31)	28.02 (1.33)	0.00931	0.33
rG_insular_short	28.17 (2.87)	27.21 (2.89)	0.0292	0.014
lS_circular_insula_sup	29.20 (1.66)	28.69 (1.53)	0.0353	0.078
rG_Ins_lg_and_S_cent _ins	26.87 (3.06)	26.06 (3.27)	0.0999	0.46
rG_pariet_inf-Angular	28.94 (1.30)	29.35 (1.25)	0.0366	0.71
rLat_Fis-post	29.81 (1.43)	29.48 (1.55)	0.152	0.21
lG_temp_sup-Lateral	28.45 (1.28)	28.01 (1.55)	0.0455	0.27
lS_pericallosal	33.64 (2.13)	32.72 (2.86)	0.0188	0.38
rG_front_middle	29.17 (1.11)	29.33 (1.06)	0.345	0.99
lLat_Fis-post	30.26 (1.80)	30.01 (1.34)	0.315	0.21

*P*-value is adjusted for age and sex.

HC, healthy control; MDD, major depressive disorder; VIP, variable importance in projection.

### Relationship between important features of differentially expressed LGIs and plasma BDNF levels

We showed the relationship between differentially expressed LGIs and plasma BDNF levels in HCs (see Supplementary Table 2) and patients with MDD (see Supplementary Table 3). We examined the correlation between plasma BDNF levels and 10 differentially expressed LGIs (VIP > 1.5 and *p*-value adjusted for age and sex < 0.05) using age and sex as confounding factors. However, we found no significant correlation between the LGIs and plasma BDNF at any location. In addition, BDNF showed no significant interaction between MDD and HC groups for the 10 LGIs (see Supplementary Table 4).

### Correlation between HAM-D and LGI

We also examined the top 10 LGIs (VIP > 1.5 and *p*-value adjusted for age and sex < 0.05) and psychiatric symptoms using these top features. We found a statistically significant correlation between rG_temp_sup-Plan_polar and HAM-D (standard partial regression coefficient = 0.238, *p* = 0.049); however, no significant correlations were observed between LGI and HAM-D total scores after Bonferroni correction (*p* = 0.49) (see Supplementary Table 5).

## Discussion

This study provides evidence of cortical gyrification pattern differences between first-episode, drug-naïve MDD patients and HCs. In this study, we found that 108 (71.0%) of the 152 total LGIs were MDD < HC. We then identified 10 disease-differentiating factors based on critical LGIs features (VIP > 1.5 and *p*-value adjusted for age and sex < 0.05), but found no significant correlation between LGIs and plasma BDNF level at any location. In addition, BDNF level showed no significant interaction between MDD and HC groups for the 10 determined LGIs.

Several previous studies have reported altered LGI in patients with MDD compared to HCs, including decreased LGI in the bilateral precuneus (23), bilateral mid-posterior cingulate, insula, orbital frontal cortices (orbitofrontal cortex), left anterior cingulate cortex (right anterior cingulate gyrus cortex), right temporal operculum (27), left lingual gyrus (left lingual corpuscle), right posterior superior temporal sulcus (right superior temporal sulcus) (29) and precuneus, superior parietal gyrus, parahippocampal gyrus, middle frontal gyrus, and fusiform and right fusiform gyrus (46). There are also several reports supporting increased LGI in patients with MDD, including increased LGI in the frontal, cingulate, parietal, temporal, and occipital regions (9); right rostral anterior cingulate cortex and medial orbitofrontal cortex; frontal pole (28); left anterior cingulate; right precentral, supramarginal region; and left superior frontal gyrus (47). However, in this study, we found that 108 (71.0%) of the 152 LGIs were MDD < HC, supporting previous findings of a decreased LGI in patients with MDD.

Some studies have reported associations between cortical gyrification patterns and BDNF level. Cortical gyrification refers to the forming process of cerebral cortex folds. During the third trimester, the brain develops from a relatively smooth erect brain structure to one that more closely resembles adult brain morphology. LGI, a measure of the degree of cortical folding, increases dramatically during the third trimester of pregnancy but remains relatively constant throughout the rest of development (48). LGI peaks by age 2 and then gradually decreases with increasing age (15, 49). One study prospectively followed patients with bipolar disorder for 4 years and found that those with one or more BDNF Met alleles, indicating that decreased BDNF secretion produced a more significant reduction in LGI (36). Besides, the neuroplasticity in brain formation—including cortical gyrification in fetal sheep—is more critical in brain regions rich in BDNF expression (50).

Astrogenesis, gliogenesis, and angiogenesis may also be related to cortical gyrification. A recent study reported that localized astrogenesis plays an important role in gyrus formation in the gyrencephalic cerebral cortex. In functional genetic experiments using ferrets, reducing astrocyte numbers prevents gyrus formation in the cortex. Meanwhile, increasing astrocyte numbers in mice, which do not have cortical folds, can induce gyrus-like protrusions (51). Gliogenesis is also involved in the gyrification of the primate cerebrum (52). In summary, astrocytes—one of the microglia—might play a role in cortical gyrification. Fibroblast growth factor 2, which is a member of a large family of proteins that bind heparin and heparan sulfate and modulate the function of a wide range of cell types, stimulates the growth and development of angiogenesis, thus contributing to the pathogenesis of several diseases including atherosclerosis, and is associated with cortical gyrification in the mouse brain (53). Taken together, astrogenesis, gliogenesis, and angiogenesis, whereas, neurogenesis might be associated with cortical gyrification in the brain.

Microglial activation plays a role in maintaining the delicate balance of BDNF release into neuronal synapses (54). Thus, BDNF secretion is considered to be influenced by astrocytes and glia, which might influence cortical gyrification. We did not, however, find an association between plasma BDNF level and LGI in this study. Therefore, plasma BDNF level may be independent of cortical gyrification. Additionally, it is controversial that plasma BDNF level reflects BDNF dynamics in the brain. These findings indicate that the association between cortical gyrification and BDNF level must still be further elucidated.

In addition, we focused on the association between gyrification patterns and depression severity. One study adjusted for age, sex, education level, and total cortical surface area reported positive correlations between LGI in the left caudal middle frontal cortex and Beck Depression Inventory scores (47). Another recent study examined correlations between the HAM-D and LGI in 22 cortical regions of patients with MDD but found no significance in the field (28). Yet another previous study reported an inverse correlation between LGI and HAM-D scores in the right inferior parietal, right postcentral, and left superior parietal lobes of patients with MDD (9). We also assessed the correlation between the top 10 gyrification patterns and psychiatric symptoms; however, we found no statistically significant correlations between LGI and HAM-D total scores.

This study had several limitations. First, the number of participants was small, and the age and sex distributions of the two groups were significantly different—a typical limitation of clinical research. In addition, we did not include education level or dominant arm in the analysis because of missing data. This may have resulted in insufficient statistical power to confirm our hypothesis. However, these differences in age and educational level may be somewhat acceptable considering that LGI peaks by age 2 and then remains relatively constant throughout one’s life. In addition, we also statistically calculated age and sex-adjusted *p*-value. Due to the limited sample size, the statistical analysis also had several limitations. We could not replicate the results to determine the validity of the outcome. However, *a priori* dimensionality reduction techniques such as PCA and OPLS-DA were used to refine the variables in examining differences in gyration patterns between the MDD and HC groups, thus increasing the accuracy of the analysis. Another point to consider is that we conducted this study using a one-point cross-sectional method. Thus, whether there were any changes in LGI during the progression of MDD was not fully elucidated. Therefore, a longitudinal study is warranted in the future.

## Conclusion

Predicated on previous publications, this is not the first study that investigated differences in cortical gyrification patterns between MDD and HC groups. The present study observed several gyrification patterns that cause differences in the disease predicated on essential features of and VIP scores and adjusted *p*-value. Of the 152 LGIs measured in this study, 108 were MDD < HC. Future large-scale studies with more participants would help to clarify the relationship between gyrification patterns and psychiatric symptoms.

## Data availability statement

The raw data supporting the conclusions of this article will be made available by the authors, without undue reservation.

## Ethics statement

The studies involving human participants were reviewed and approved by the Ethical Committee of University of Occupational and Environmental Health, Japan. The patients/participants provided their written informed consent to participate in this study.

## Author contributions

TN, NO, KW, and RY conceived and designed the experiments. TN, NO, EC, HT, KW, and RY performed the experiments. TN, NO, EC, and KW analyzed the data. TN, NO, KW, EC, and RY composed the manuscript. TN, NO, EC, KW, HT, GH, AI, SK, and RY provided expertise and edited the manuscript. All authors read the manuscript and were solely and jointly responsible for its content.

## Footnotes

1
https://www.fil.ion.ucl.ac.uk/spm

2
http://dbm.neuro.uni-jena.de/cat/

3
http://www.neuro.uni-jena.de/cat12/CAT12-Manual.pdf
