# Supplementary material for: Gyrification patterns in first-episode, drug-naïve major depression: Associations with plasma levels of brain-derived neurotrophic factor and psychiatric symptoms
Source: Front Psychiatry. 2023 Jan 6;13:1031386. doi: 10.3389/fpsyt.2022.1031386 (PMC9852994; doi:10.3389/fpsyt.2022.1031386)
Supplement: Supplementary file 1 [file Data_Sheet_1.pdf]

Supplementary Table 1. LGI in MDD and HC groups

	HC		MDD	
	Left	Right	Left	Right
Unknown	23.81[22.84 - 24.67]	23.41 [22.63 - 24.27]	22.93 [21.93 - 24.07]	22.76 [21.89 - 23.64]
G_and_S-frontomargin	31.31 [30.57 - 32.34]	32.36 [31.06 - 33.23]	31.75 [30.83 - 32.44]	31.97 [31.24 - 33.68]
G_and_S_occipital_inf	30.05 [29.12 - 31.06]	30.76 [29.37 - 31.72]	30.35 [29.05 - 31.50]	30.58 [29.72 - 31.43]
G_and_S_paracentral	26.75 [25.86 - 27.49]	27.39 [26.11 - 28.49]	26.66 [25.83 - 27.23]	27.11 [26.02 - 28.13]
G_and_S_subcentral	28.68 [27.84 - 29.58]	28.68 [27.52 - 29.78]	28.48 [27.41 - 29.35]	28.46 [27.60 - 29.42]
G_and_S_transv_frontopol	32.00 [30.19 - 32.91]	31.85 [30.60 - 32.91]	31.49 [30.64 - 32.63]	31.71 [30.71 - 32.88]
G_and_S_cingul-Ant	30.06 [28.83 - 30.79]	29.91 [29.12 - 30.72]	29.64 [28.93 - 30.47]	29.56 [28.57 - 30.33]
G_and_S_cingul-Mid-Ant	27.88 [26.90 - 29.24]	28.35 [27.67 - 29.41]	27.67 [26.17 - 28.73]	28.16 [27.24 - 29.53]
G_and_S_cingul-Mid-Post	29.26 [28.04 - 30.17]	28.74 [27.93 - 29.79]	28.89 [27.77 - 29.94]	28.65 [27.79 - 29.67]
G_cingul-Post-dorsal	31.37 [29.94 - 32.77]	31.14 [29.84 - 32.46]	30.09 [28.93 - 32.24]	30.85 [30.15 - 32.52]

G_cingul-Post-ventral	29.11 [26.51 - 30.65]	27.62 [25.76 - 29.44]	27.08 [23.82 - 30.01]	26.41 [24.08 - 28.64]
G_cuneus	30.71 [29.58 - 31.76]	31.27 [30.35 - 32.19]	30.79 [29.79 - 31.92]	30.94 [29.98 - 31.85]
G_front_inf-Opercular	26.81 [25.67 - 27.78]	27.12 [26.16 - 28.04]	26.30 [25.70 - 27.54]	27.05 [25.91 - 28.12]
G_front_inf-Orbital	29.02 [27.27 - 30.78]	29.51 [27.71 - 30.63]	28.39 [26.95 - 30.36]	29.87 [28.52 - 31.81]
G_front_inf-Triangul	28.80 [27.65 - 29.71]	29.17 [28.13 - 29.90]	28.29 [27.16 - 29.43]	28.86 [27.99 - 29.94]
G_front_middle	29.10 [28.46 - 29.88]	29.26 [28.43 - 29.98]	29.22 [28.49 - 29.81]	29.30 [28.53 - 30.05]
G_front_sup	27.90 [27.15 - 28.44]	27.97 [27.18 - 28.69]	27.80 [27.18 - 28.21]	27.84 [27.26 - 28.42]
G_Ins_lg_and_S_cent_ins	27.80 [26.67 - 29.29]	27.13 [24.79 - 29.05]	27.31 [25.80 - 28.77]	26.36 [24.06 - 27.90]
G_insular_short	27.91 [26.52 - 29.19]	28.35 [26.78 - 30.21]	27.92 [26.22 - 29.99]	27.15 [24.99 - 29.15]
G_occipital_middle	30.99 [30.18 - 31.67]	30.81 [30.29 - 31.76]	31.00 [30.17 - 31.76]	30.90 [30.09 - 31.62]
G_occipital_sup	30.54 [29.61 - 31.46]	29.50 [28.73 - 30.62]	30.10 [29.39 - 30.92]	29.68 [28.66 - 30.60]
G_oc-temp_lat-fusifor	28.57 [27.38 - 29.81]	28.52 [27.54 - 29.85]	28.25 [27.18 - 28.97]	28.64 [27.48 - 29.82]
G_oc-temp_med-Lingual	31.11 [30.16 - 31.96]	30.55 [29.26 - 31.47]	30.40 [29.65 - 31.68]	30.39 [29.27 - 31.21]

G_oc-temp_med-Parahip	25.78 [24.87 - 26.69]	26.22 [25.14 - 27.35]	24.41 [23.81 - 25.20]	24.91 [24.19 - 25.94]
G_orbital	29.88 [28.85 - 30.65]	29.34 [28.73 - 30.23]	29.56 [28.55 - 30.61]	29.27 [28.22 - 30.03]
G_pariet_inf-Angular	28.95 [28.11 - 29.80]	29.18 [28.03 - 29.91]	29.19 [28.44 - 29.99]	29.40 [28.69 - 29.98]
G_pariet_inf-Supramar	28.21 [27.64 - 29.02]	28.29 [27.54 - 28.91]	28.10 [27.22 - 28.66]	28.06 [27.40 - 28.67]
G_parietal_sup	28.51 [27.66 - 29.29]	28.43 [27.65 - 29.57]	28.58 [27.96 - 29.31]	28.44 [27.41 - 29.32]
G_postcentral	27.46 [26.47 - 28.27]	26.65 [25.90 - 27.84]	26.86 [26.24 - 27.56]	26.57 [25.37 - 27.78]
G_precentral	24.19 [23.34 - 25.51]	23.78 [23.02 - 24.97]	24.05 [23.18 - 24.65]	23.84 [22.99 - 24.62]
G_precuneus	30.02 [29.02 - 30.92]	30.00 [28.98 - 30.68]	29.89 [29.09 - 30.72]	29.93 [29.36 - 30.51]
G_rectus	29.77 [28.35 - 31.31]	27.98 [26.89 - 28.74]	29.83 [28.61 - 31.52]	28.08 [27.03 - 29.17]
G_subcallosal	29.11 [25.39 - 33.27]	29.23 [27.03 - 31.70]	28.09 [26.03 - 32.46]	29.36 [26.98 - 32.38]
G_temp_sup-G_T_transv	24.78 [22.92 - 26.47]	24.79 [22.67 - 26.60]	23.74 [21.73 - 25.73]	24.01 [22.04 - 25.83]
G_temp_sup-Lateral	28.41 [27.61 - 29.28]	27.86 [26.75 - 28.80]	27.87 [26.77 - 29.21]	27.10 [26.43 - 28.09]
G_temp_sup-Plan_polar	22.36 [20.24 - 24.53]	23.54 [21.50 - 25.96]	20.89 [19.25 - 21.97]	21.61 [20.24 - 23.98]

G_temp_sup-Plan_tempo	23.93 [22.75 - 24.98]	23.56 [22.57 - 24.95]	23.08 [21.66 - 24.49]	23.01 [22.01 - 24.45]
G_temporal_inf	28.67 [27.99 - 29.29]	28.45 [27.77 - 29.12]	28.64 [27.59 - 29.55]	28.50 [27.81 - 29.43]
G_temporal_middle	28.27 [27.89 - 29.13]	28.08 [27.38 - 28.56]	28.14 [27.58 - 28.72]	27.66 [27.16 - 28.48]
Lat_Fis-ant-Horizont	25.80 [24.65 - 27.37]	24.83 [22.99 - 26.67]	26.16 [24.44 - 28.19]	24.49 [22.50 - 25.95]
Lat_Fis-ant-Vertical	26.82 [25.33 - 28.36]	26.99 [25.23 - 29.60]	26.45 [24.53 - 28.26]	27.21 [24.96 - 28.89]
Lat_Fis-post	30.42 [29.38 - 31.59]	30.04 [29.37 - 30.60]	29.95 [29.36 - 30.91]	29.76 [28.52 - 30.52]
Pole_occipital	32.46 [31.85 - 33.49]	32.70 [31.64 - 33.28]	32.60 [31.92 - 33.06]	32.12 [31.60 - 33.14]
Pole_temporal	28.53 [27.73 - 29.39]	28.73 [27.75 - 29.57]	27.94 [27.22 - 28.81]	28.41 [27.42 - 29.23]
S_calcarine	29.66 [28.80 - 30.46]	29.97 [29.16 - 30.88]	29.47 [28.69 - 30.26]	30.04 [29.23 - 30.87]
S_central	26.60 [25.94 - 27.74]	26.45 [25.52 - 27.43]	27.06 [26.15 - 27.99]	26.58 [25.40 - 27.46]
S_cingul-Marginalis	27.54 [26.05 - 28.86]	28.27 [26.72 - 30.18]	27.35 [25.55 - 29.11]	28.63 [26.64 - 30.29]
S_circular_insula_ant	27.82 [26.16 - 28.94]	28.82 [27.09 - 30.09]	27.22 [25.98 - 28.98]	27.85 [26.68 - 29.37]
S_circular_insula_inf	25.93 [24.44 - 27.54]	25.13 [23.77 - 26.15]	24.30 [22.46 - 25.20]	23.74 [21.81 - 25.17]
S_circular_insula_sup	29.35 [28.45 - 30.22]	29.69 [28.44 - 30.61]	28.80 [27.98 - 29.57]	29.14 [27.85 - 29.94]

S_collat_transv_ant	29.35 [27.96 - 30.67]	28.06 [27.42 - 29.20]	28.70 [27.39 - 29.99]	27.68 [26.77 - 29.49]
S_collat_transv_post	32.55 [30.79 - 34.30]	31.81 [30.29 - 33.55]	32.93 [31.20 - 34.47]	32.47 [30.48 - 33.61]
S_front_inf	29.01 [28.11 - 29.96]	29.19 [28.17 - 29.87]	29.10 [28.09 - 30.16]	29.34 [28.18 - 30.09]
S_front_middle	31.17 [30.23 - 32.05]	31.12 [30.37 - 32.12]	31.44 [29.78 - 32.28]	31.07 [30.15 - 31.90]
S_front_sup	28.29 [27.15 - 29.06]	27.97 [26.76 - 28.54]	28.48 [27.64 - 29.58]	28.01 [27.12 - 28.72]
S_interm_prim-Jensen	29.69 [27.68 - 31.72]	28.59 [27.03 - 30.60]	29.86 [27.69 - 32.29]	28.88 [27.01 - 30.63]
S_intrapariet_and_P_trans	29.81 [28.49 - 30.63]	28.83 [27.69 - 30.34]	29.56 [28.48 - 30.59]	29.01 [27.84 - 30.08]
S_oc_middle_and_Lunatus	31.81 [30.71 - 33.08]	31.19 [29.53 - 32.18]	31.98 [30.74 - 33.27]	31.59 [29.27 - 32.82]
S_oc_sup_and_transversal	31.24 [30.36 - 32.09]	31.13 [30.09 - 32.12]	31.44 [29.94 - 32.09]	31.28 [30.18 - 32.26]
S_occipital_ant	29.79 [28.00 - 31.23]	30.36 [28.63 - 32.10]	30.46 [28.68 - 31.61]	30.54 [28.73 - 32.27]
S_oc-temp_lat	31.04 [29.98 - 32.19]	30.82 [29.27 - 32.08]	31.24 [29.99 - 32.62]	31.36 [29.88 - 32.41]
S_oc-temp_med_and_Lingual	28.70 [27.34 - 30.02]	27.49 [26.28 - 28.52]	28.92 [27.27 - 29.66]	27.28 [26.41 - 29.05]

S_orbital_lateral	30.53 [29.21 - 31.87]	31.25 [29.54 - 33.18]	31.18 [29.78 - 32.73]	31.59 [29.86 - 32.97]
S_orbital_med-olfact	30.66 [29.32 - 32.78]	31.40 [29.36 - 32.98]	30.55 [28.12 - 32.70]	30.15 [28.87 - 31.86]
S_orbital-H_Shaped	31.00 [29.78 - 32.03]	31.17 [30.00 - 31.98]	30.94 [30.18 - 32.10]	30.86 [29.71 - 31.85]
S_parieto_occipital	29.87 [29.17 - 30.89]	29.77 [28.82 - 30.66]	29.69 [28.75 - 30.45]	29.67 [28.28 - 30.53]
S_pericallosal	33.88 [32.50 - 34.98]	34.10 [33.19 - 35.23]	32.77 [31.34 - 34.68]	34.10 [32.60 - 34.99]
S_postcentral	28.26 [26.98 - 29.34]	28.01 [26.47 - 29.49]	28.22 [26.76 - 29.18]	27.63 [26.62 - 28.81]
S_precentral-inf-part	27.28 [26.27 - 28.21]	27.33 [26.17 - 28.38]	27.27 [26.44 - 28.50]	27.51 [26.40 - 28.55]
S_precentral-sup-part	27.24 [25.86 - 28.09]	27.00 [25.38 - 27.98]	27.47 [26.03 - 28.47]	26.88 [25.97 - 27.95]
S_suborbital	30.36 [28.08 - 32.80]	33.17 [29.22 - 35.71]	30.00 [27.94 - 32.53]	32.96 [31.05 - 34.44]
S_subparietal	32.21 [30.88 - 33.11]	31.89 [30.95 - 32.82]	31.87 [30.99 - 33.16]	32.03 [30.87 - 32.83]
S_temporal_inf	30.47 [29.56 - 31.40]	30.62 [29.48 - 31.64]	30.34 [29.07 - 31.47]	30.08 [29.09 - 31.43]
S_temporal_sup	27.94 [27.13 - 28.66]	27.27 [26.69 - 28.14]	27.52 [26.91 - 28.55]	27.29 [26.33 - 27.98]
S_temporal_transverse	28.87 [27.82 - 30.01]	28.03 [25.61 - 29.69]	28.28 [26.56 - 29.64]	26.93 [24.01 - 29.01]

Supplementary Table 2. Relationship between differentially expressed gyrification and plasma levels of BDNF in HCs

	Standard partial regression coefficient	95 % C. I	Standard error	t value	Adjusted p-value
rS_circular_insula_inf	0.063	-0.172 ~ 0.299	0.117	0.539	0.59
lUnknown	0.183	-0.021 ~ 0.387	0.101	1.802	0.077
lS_circular_insula_inf	0.064	-0.141 ~ 0.269	0.102	0.627	0.53
rUnknown	0.012	-0.174 ~ 0.199	0.092	0.133	0.89
lG_oc-temp_med-Parahip	0.029	-0.229 ~ 0.287	0.128	0.226	0.82
lG_temp_sup-Plan_polar	-0.058	-0.313 ~ 0.197	0.127	-0.458	0.64
rG_oc-temp_med-Parahip	0.080	-0.113 ~ 0.274	0.096	0.831	0.41

rG_temp_sup-Plan_polar	0.047	-0.215 ~ 0.311	0.130	0.363	0.71
lG_cingul-Post-ventral	-0.072	-0.344 ~ 0.199	0.135	-0.533	0.59
rG_insular_short	0.023	-0.185 ~ 0.232	0.104	0.227	0.82

P-value is adjusted for age and sex.

Supplementary Table 3. Relationship between differentially expressed gyrification and plasma levels of BDNF in patients with MDD

	Standard partial regression coefficient	95 % C. I	Standard error	t value	Adjusted p-value
rS_circular_insula_inf	-0.060	-0.412 ~ 0.291	0.171	-0.354	0.72
lUnknown	0.120	-0.148 ~ 0.389	0.130	0.923	0.36

lS_circular_insula_inf	-0.124	-0.536 ~ 0.287	0.200	-0.620	0.54
rUnknown	-0.021	-0.320 ~ 0.277	0.145	-0.148	0.88
lG_oc-temp_med-Parahip	0.003	-0.391 ~ 0.398	0.192	0.019	0.98
lG_temp_sup-Plan_polar	-0.097	-0.461 ~ 0.266	0.177	-0.549	0.58
rG_oc-temp_med-Parahip	-0.103	-0.558 ~ 0.351	0.221	-0.466	0.64
rG_temp_sup-Plan_polar	-0.010	-0.383 ~ 0.363	0.181	-0.057	0.95
lG_cingul-Post-ventral	0.238	-0.152 ~ 0.628	0.189	1.255	0.22
rG_insular_short	-0.063	-0.357 ~ 0.229	0.142	-0.447	0.65

P-value is adjusted for age and sex.

Supplementary Table 4. Interaction analysis between gyrification in both groups (MDD, HC) and BDNF.

	Standard partial regression coefficient	95 % C. I	Standard error	t value	Adjusted p-value
rS_circular_insula_inf	-0.112	-0.480 ~ 0.255	0.184	-0.608	0.54
lUnknown	-0.0674	-0.377 ~ 0.242	0.155	-0.433	0.66
lS_circular_insula_inf	-0.153	-0.533 ~ 0.225	0.190	-0.809	0.42
rUnknown	-0.0453	-0.347 ~ 0.257	0.151	-0.299	0.76
lG_oc-temp_med-Parahip	-0.020	-0.424 ~ 0.384	0.202	-0.099	0.92
lG_temp_sup-Plan_polar	-0.013	-0.408 ~ 0.381	0.198	-0.067	0.94
rG_oc-temp_med-Parahip	-0.165	-0.533 ~ 0.202	0.184	-0.897	0.37

rG_temp_sup-Plan_polar	-0.061	-0.468 ~ 0.345	0.204	-0.301	0.76
lG_cingul-Post-ventral	0.300	-0.114 ~ 0.715	0.208	1.443	0.15
rG_insular_short	-0.058	-0.384 ~ 0.268	0.163	-0.354	0.72

P-value is adjusted for age and sex.

Supplementary Table 5. Relationship between differentially expressed gyrification and HAM-D total scores in patients with MDD

	Standard partial regression coefficient	95 % C. I	Standard error	t value	Adjusted p-value
rS_circular_insula_inf	0.148	-0.137 ~ 0.434	0.143	1.036	0.30
lUnknown	0.00106	-0.304 ~ 0.306	0.153	0.00693	0.99

lS_circular_insula_inf	0.149	-0.110 ~ 0.409	0.130	1.148	0.25
rUnknown	0.0595	-0.247 ~ 0.366	0.154	0.386	0.70
lG_oc-temp_med-Parahip	0.010	-0.222 ~ 0.244	0.117	0.091	0.92
lG_temp_sup-Plan_polar	-0.021	-0.290 ~ 0.248	0.135	-0.157	0.87
rG_oc-temp_med-Parahip	-0.165	-0.407 ~ 0.075	0.121	-1.367	0.17
rG_temp_sup-Plan_polar	0.241	0.00015 ~ 0.483	0.121	1.993	0.049
lG_cingul-Post-ventral	0.066	-0.169 ~ 0.301	0.118	0.559	0.57
rG_insular_short	0.087	-0.160 ~ 0.334	0.124	0.700	0.48

P-value is adjusted for age and sex.
